# Efficacy and safety of intraventricular nimodipine in patients with aneurysmal subarachnoid hemorrhage compared to oral nimodipine: a systematic review and meta-analysis

**DOI:** 10.1007/s10143-025-03563-5

**Published:** 2025-05-19

**Authors:** Hesham Kelani, Nancy Ibrahim, Ahmed Naeem, Hazem Mohamed Salamah, Nada Ashraf Al-Shafey, Yousef Tanas, Esraa Shawky Ibrahem, Fawzy Mohamed Fawzy Naga, Volodymyr Vulkanov, Mohamed Jadidi, David P. Lerner, Ketevan Berekashvili, Moshe A. Mizrahi, Alexander Andreev, Diana Greene-Chandos, Athos Patsalides

**Affiliations:** 1 Neurology Department, SUNY Downstate at One Brooklyn Health, Brooklyn, NY USA; 2 Faculty of Medicine, Alexandria University, Alexandria, Egypt; 3 Al-Azhar Faculty of Medicine, Asyut, Egypt; 4 Faculty of Medicine, Zagazig University, Zagazig, Egypt; 5 Department of Neurosurgery, Rutgers New Jersey School of Medicine, Newark, NJ USA; 6 Department of Neurology, Zucker School of Medicine, Northwell health, Hempstead, NY USA; 7 Department of Neurology, School of Medicine, University of Saint Louis, Saint Louis, MO USA; 8 Department of Neurosurgery, North Shore University Hospital, Northwell, Manhasset, NY USA

**Keywords:** Nimodipine, Meta-analysis, Delayed cerebral ischemia, Subarachnoid hemorrhage, Vasospasm

## Abstract

Aneurysmal subarachnoid hemorrhage (aSAH) is associated with high morbidity and mortality. Nimodipine is the only drug approved and administered orally as well as intravenously to improve the outcomes of patients with vasospasm post-SAH. EG-1962 is a sustained-release formulation of nimodipine administered into the subarachnoid space in patients with aSAH. We hypothesize that this may improve the efficacy of nimodipine and minimize its adverse effects. We searched PubMed, Cochrane, Scopus, and Web of Science on August 2, 2024, using relevant keywords. Studies were screened for eligibility. We extracted the data from the relevant articles and then these data were pooled as risk ratio (RR) and 95% confidence interval (CI), using R software. Pooled data from trials comparing intraventricular nimodipine with oral nimodipine showed a significantly lower risk of angiographic vasospasm in the intraventricular nimodipine cohort than the oral nimodipine cohort (RR = 0.8, CI [0.65–0.98]), and a trend towards lower risk of delayed cerebral ischemia and hypotension. No significant difference in extended Glasgow coma scale (eGCS) at 90 days of follow-up and other adverse events like hydrocephalus, bacterial meningitis, and serious adverse events including death. The risk of angiographic vasospasm was lower with intraventricular nimodipine compared with oral nimodipine, and there was a trend toward a decreased incidence of DCI and hypotension, which should be validated in future studies. However, there is no significant improvement in functional outcomes from intraventricular nimodipine. More rigorous research is needed to look at the underlying mechanisms and see if other factors influence the functional outcomes.

**Clinical trial number** Not applicable.

**Supplementary Information:**

The online version contains supplementary material available at 10.1007/s10143-025-03563-5.

## Introduction

Spontaneous aneurysmal subarachnoid hemorrhage (aSAH) is a life-threatening condition that occurs in 5 to 10 per 100,000 people annually [1]. aSAH is a severe type of hemorrhagic stroke with a mortality rate of up to 35% and more than one third of survivors being disabled and functionally dependent [2–4]. The global crude incidence of aSAH is 7.9 per 100,000 person-years (95% CI, 6.9–9.0) [5]. Women had a 30% higher risk than men, especially those over 75 years, particularly in Japan and to a lesser extent in Europe. There is an annual reduction of 1.7% (95% CI, 0.6–2.8) between 1955 and 2014. Regionally, incidence dropped substantially in Europe (40.6%), Asia (46.2%), and North America (14.0%), but rose sharply in Japan by 59.1%. Severe complications result from aSAH, including acute rebleeding [6, 7], acute hydrocephalus [1], CNS infection [2] and delayed cerebral ischemia (DCI) [1].

DCI is a major contributor to poor outcomes in approximately 70% of the patients [8] and death in up to 30% of patients with aSAH [9]. DCI is a specific subtype of ischemic stroke that occurs one week after aneurysmal rupture [10]. The mechanism of DCI has been widely recognized as a multifactorial complication that cannot be explained by cerebral arterial vasospasm alone [10]. Traditionally, cerebral arterial vasospasm resulting from vasoactive mediators released from extravasated blood has been considered the major mechanism of DCI [11]. A reduced level of the vasodilator peptide calcitonin gene-related peptide (CGRP) from the trigeminal afferents has been observed in cerebral nerves and blood vessels after aSAH, leading to blood vessel vasoconstriction and vasospasm [12, 13]. However, some trials reported no significant clinical improvement in patients with aSAH following treatment with the endothelin A receptor antagonist clazosentan, which caused a 65% relative risk reduction of angiographic vasospasm [14, 15]. Heterogeneous perfusion patterns in positron emission studies suggest that the mechanism of DCI is more dynamic than angiographic vasospasm [16]. Some studies reported an association between angiographic vasospasm and cerebral perfusions [11, 17]. Furthermore, other factors could lead to DCI, including microthrombosis [1, 18], volume contraction, oxygen free radicals, hyponatremia, bilirubin oxidation products, chemical factors released from the clot like local potassium or hemoglobin, spreading ischemia, and endothelin-1 or nitric oxide [10]. Spreading depolarization occurs frequently after aSAH and is closely associated with spreading ischemia and DCI [19].

Spreading depolarization (SD), resulting from ischemia, is the underlying mechanism of cytotoxic edema of the brain gray matter [19]. SD describes the neural tissue depolarization wave with disruption of ion concentration gradients and neural membrane resistance, resulting in electrical activity loss, swelling, and distortion of the dendritic spines [20].

As a result of the ubiquity of L-type calcium channels in multiple cell types, nimodipine, a dihydropyridine calcium channel antagonist, has a possible beneficial effect in the prophylaxis and treatment of delayed cerebral ischemia resulting from aSAH through synergistic effects on neural tissue, glial cells, and vascular pathways [8, 21, 22]. Nimodipine, administered orally or intravenously, is used in cerebrovascular disorders to induce vasorelaxation by inhibiting calcium influx into the vascular smooth muscle through L-type calcium channels, as well as to achieve a neuroprotective effect by preventing neuronal calcium overload [22]. However, systemic nimodipine use is limited due to its potential side effects [8]. Hypotension, a well-known side effect of systemic nimodipine, has a deleterious effect on patients with aSAH through decreasing cerebral perfusion pressure, which in turn may worsen the risk of DCI [23, 24]. Despite the high levels of nimodipine plasma concentrations, the cerebrospinal fluid concentration remains below the therapeutic threshold [25]. The recommended dose of nimodipine for SAH patients is 60 mg orally every 4 h for 21 days post aSAH; however, oral administration is limited by poor and variable oral bioavailability, difficult dose compliance, vertigo, flushing, bradycardia, pedal edema, and death in some cases [26]. A single injection of a sustained-release formulation of EG-1962 (a biodegradable polymer that releases nimodipine for at least 21 days after injection) into the subarachnoid space near the cerebral arteries is thought to increase the efficacy and overcome the limitations of systemic nimodipine dosing, such as hypotension, and the difficult dose regimen compliance [27, 28]. Some studies reported that intraventricular nimodipine is superior to oral nimodipine [29]. On the other hand, other studies showed no significant improvement in the favorable outcomes [14].

No previous systematic review or meta-analysis was done to evaluate the evidence regarding the efficacy and safety of intraventricular nimodipine compared to the oral route. Here, we conducted the first systematic review and meta-analysis to compare intraventricular nimodipine to oral nimodipine based on their efficacy and safety profiles.

## Methodology

This article was prepared following the Preferred Reporting Items for Systematic Reviews and Meta-Analysis (PRISMA) Statement Guidelines [30]. PRISMA checklist can be found in the supplementary material. The protocol for this systematic review was registered on PROSPERO (International prospective register of systematic reviews) with ID CRD42024592586.

### Eligibility criteria

Published studies were included if they met the following population, intervention, comparison, outcomes, and study (PICOS) criteria: patients with aneurysmal subarachnoid hemorrhage (P); intraventricular nimodipine (I); oral nimodipine (C); any outcome observed (O); and randomized controlled trials (S).

Case reports, case series, review articles, editorial comments, RCT study protocols, animal studies, subarachnoid hemorrhage not caused by aneurysmal rupture, and nonpeer-reviewed articles were all excluded. Studies with randomized grouping after cerebral vasospasm were excluded, as in this situation, nimodipine would have no effect on prevention of cerebral vasospasm and delayed cerebral ischemia, which may affect the primary outcome, favorable functional outcome at day 90.

### Information source

A systematic search was performed in the PubMed, Scopus, Web of Science, and Cochrane databases for relevant studies through August 2, 2024. The reference lists of the included studies and of any related reviews were also checked for relevant studies.

### Search strategy

The databases were searched using the following search strategy: (Nimodipine OR Nimotop OR “calcium channel blocker” OR Dihydropyridine OR “EG-1962” OR Nymalize) AND (“subarachnoid hemorrhage*” OR SAH* OR “intracranial subarachnoid hemorrhage*” OR “Spontaneous Subarachnoid Hemorrhage” OR aSAH). No restrictions were applied to the language or the year of publication.

### Study selection

The endnote was used to combine the search results of the databases and was then exported as an Excel file. Rayyan was used for semi-automated screening of literature search results. Studies were screened in two phases. The first phase was title/abstract screening for potential clinical studies. In the second phase, the full-text articles of the selected abstracts were retrieved for further eligibility screening. The screening was done by two independent authors and any disagreement was resolved through consultation with a senior reviewer.

### Data extraction

For all included studies, data were extracted to a uniform online data extraction sheet. The extracted data were related to summary information of the included studies, such as study ID, study design, sample size, study arms, inclusion criteria, exclusion criteria, results, and conclusion; and the baseline characteristics of the included patients, including age, sex, WFNS grades, and modified Fisher scale. Data extraction was done independently by two review authors. Any disagreement was resolved through consultation with a senior reviewer.

### Outcomes

The primary outcome of interest was favorable functional outcome at day 90 as measured by the extended Glasgow coma scale (eGCS ≥ 6) among people of different World Federation of Neurological Surgeons (WFNS) grades. Secondary outcomes included angiographic vasospasm, delayed cerebral ischemia, hypotension, hydrocephalus, bacterial meningitis, and serious adverse events, such as death.

### Quality assessment

The Cochrane Risk of Bias revised tool [31] was used to evaluate the included studies. It is divided into five domains: (A) bias arising from the randomization process; (B) bias due to deviations from intended interventions; (C) bias due to missing outcome data; (D) bias in the measurement of outcomes; and (E) bias in the selection of the reported results. The domains received a risk assessment of low, some concern, or high. Two authors carried out the evaluation independently, consulting a third author where necessary.

### Statistical analysis

The R software was used for the meta-analysis using the “meta” package. Outcomes were pooled using the risk ratio (RR) with 95% confidence intervals (CIs). Heterogeneity between pooled studies was assessed by chi-square and I-square tests. The studies were considered heterogeneous at chi-square p-value < 0.1 and I² > 50%. We used the random effect model to account for the heterogeneity between the studies. If no heterogeneity was detected, we used a fixed-effect model. Due to the small number of studies in the analysis, we could not apply sensitivity analysis (leave one study out) or assess the publication bias. In general, a P value less than 0.05 was considered statistically significant.

## Results

### Literature search

**Fig. 1:**
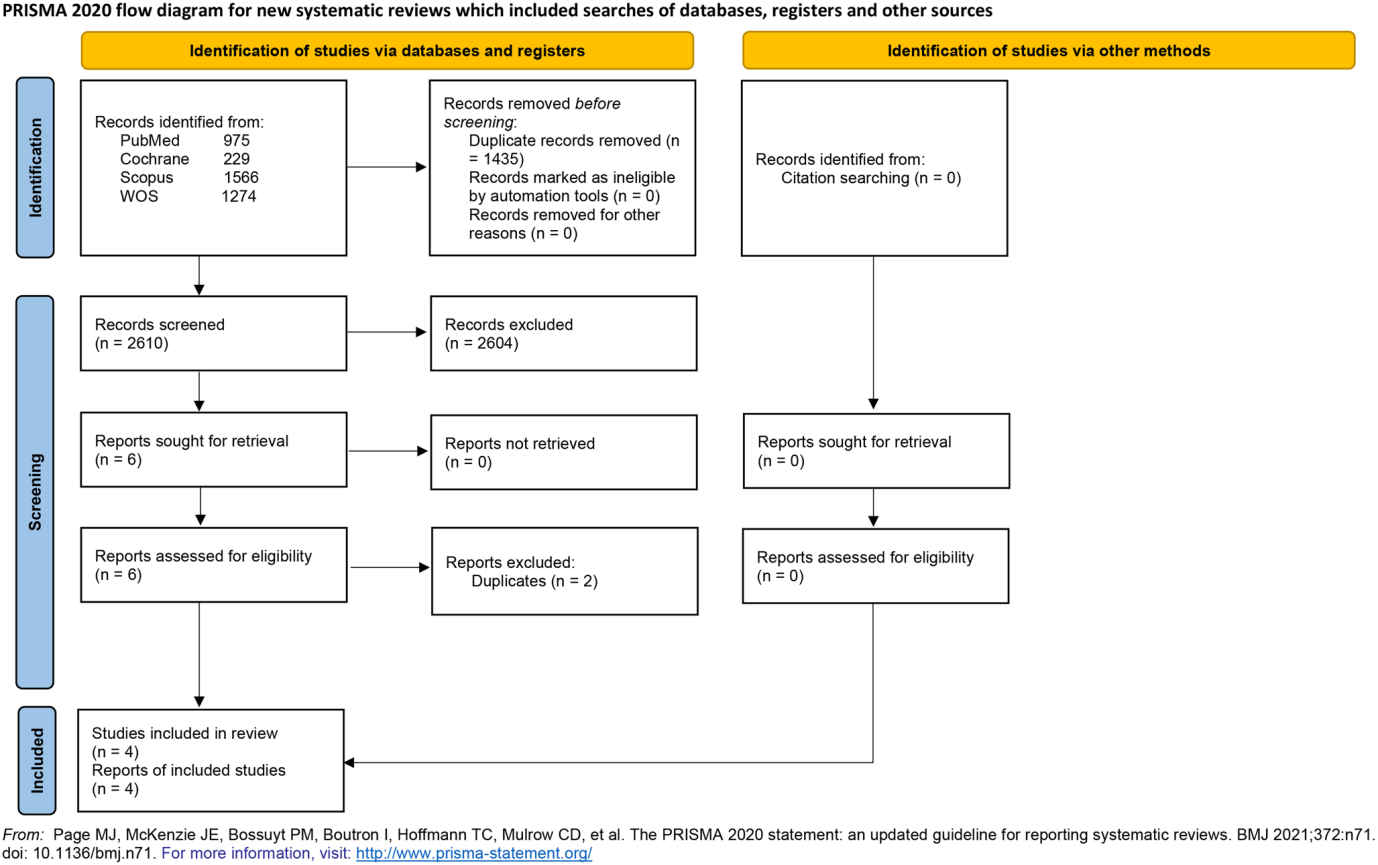
PRISMA flow diagram shows the detailed search and selection process

The literature search retrieved 4045 citations. After title and abstract screening, 6 articles were retrieved for full-text screening. Two of them were duplicates and were excluded. No missing papers were found after screening the references of included trials. Finally, four studies were included in our meta-analysis. Figure 1 shows the PRISMA flow diagram.

### Characteristics of the included studies

A summary of the included studies and baseline characteristics of their patients is shown in Table 1. The intervention group in the included papers received a single dose of 600 mg sustained-release intraventricular nimodipine plus oral placebo and the control group received the standard oral nimodipine regimen plus a single intraventricular injection of NaCl, 0.9%.

**Table 1:** Characteristic of the included patient and summery of the studies

Study ID	Study design	Characteristics of intervention and control	Study groups	Sample size	Age, {years} Mean (SD)	Sex M/F	WFNS *n* (%)			Modified fisher scale *n* (%)		
							Grade 2	Grade 3	Grade 4	Grade 2	Grade 3	Grade 4
Carlson, 2019. [1]	Double-blind, double-dummy randomized clinical study	Arm 1: Single dose of 600 mg sustained-release intraventricular EG-1962 with oral placebo. Arm 2: Single intraventricular injection of NaCl 0.9% with oral nimodipine.	Intraventricular nimodipine	144	56 (10)	45/99	71 (49)	73 (51) #		7 (6)	44 (31)	90 (63)
			Oral nimodipine	145	57 (11)	42/103	70 (48)	75 (52) #		10 (7)	45 (31)	89 (61)
Carlson, 2021. [2]	Double-blind, double-dummy randomized clinical study	Arm 1: Single dose of 600 mg sustained-release intraventricular EG-1962 with oral placebo. Arm 2: Single intraventricular injection of NaCl 0.9% with oral nimodipine.	Intraventricular nimodipine	3	54, 51, 68$	NA	2 (66.6)	1 (33)	0	2 (66.6)	NA	1 (33)
			Oral nimodipine	2	57, 69$	NA	0	1 (50)	1 (50)	0	NA	2 (100)
Hänggi, 2017. [3]	Double-blind, double-dummy randomized clinical study	Arm 1: Single dose of 600 mg sustained-release intraventricular EG-1962 Arm 2: oral nimodipine.	Intraventricular nimodipine	9^	56 (12)	6/3	2 (22.2)	0	7 (77.7)	3 (33)	0	6 (67)
			Oral nimodipine	18	56 (10)	5/13	5 (28)	2 (50)	11 (61)	2 (11)	5 (28)	11 (61)
R. Loch Macdonald, 2020. [4]	Multicenter, Randomized, Open-Label Safety Study	Arm 1: Single dose of 600 mg sustained-release intraventricular EG-1962 Arm 2: oral nimodipine.	Intraventricular nimodipine	5	47 (6)	5*	3 (60)	NA	NA	2 (40)	2 (40)	1 (20)
			Oral nimodipine	1	46	1*	0	NA	NA	1 (100)	0	0

WFNS: World federation of neurosurgical societies, SD: standard deviation, Data are presented as mean & (SD) or number & (%)

(*) all patients are females

# Data represents grade 3 + grade 4

$ ages of patients in years

^ numbers of patients in the cohort taking 600 mg intraventricular nimodipine

### Quality assessment

**Fig. 2:**
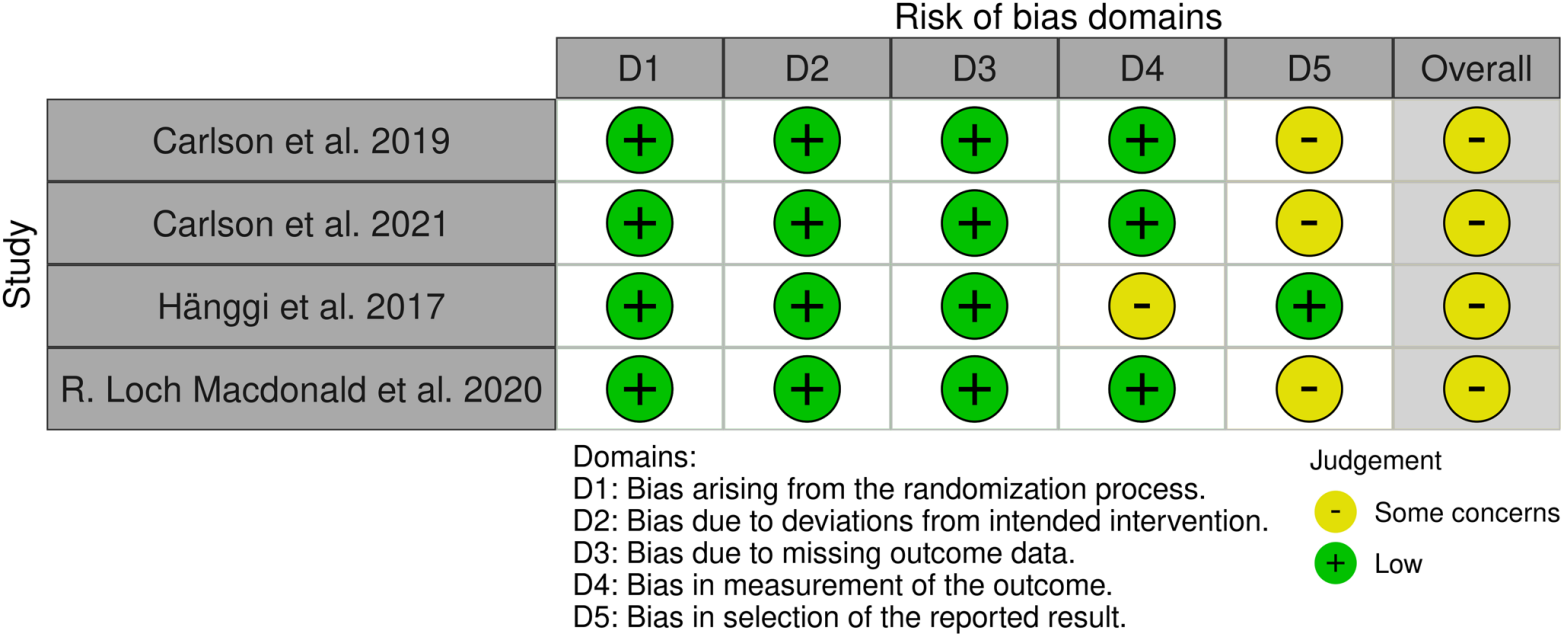
A summary of the risk of bias of the included studies

In accordance with the Cochrane tool for assessing the risk of bias (ROB2), a critical evaluation of the included randomized controlled trials (RCTs) reveals certain concerns that warrant consideration. Notably, Carlson et al. (2020) [29] raised issues regarding missing values, attributing these gaps to both true data unavailability and the reliance on intention-to-treat analysis based on final observations. Additionally, the absence of a readily available, preplanned analysis strategy casts uncertainty on the accurate assessment of outcome measures. In the case of Carlson et al. (2021) [32], the absence of a protocol introduces concerns regarding the study’s methodological transparency and rigor. Similarly, Hänggi et al. (2017) [27] lacked explicit information on assessor blinding, potentially introducing bias to the outcome assessment process. Furthermore, the trial registry record of Loch Macdonald et al. (2020) [14] failed to provide an analysis plan, thereby obscuring whether the generated data underwent evaluation based on predetermined criteria. A summary of the risk of bias assessment domains is shown in Fig. 2.

### Outcomes

#### The extended Glasgow coma scale (eGCS)

##### WFNS grade 2

**Fig. 3:**
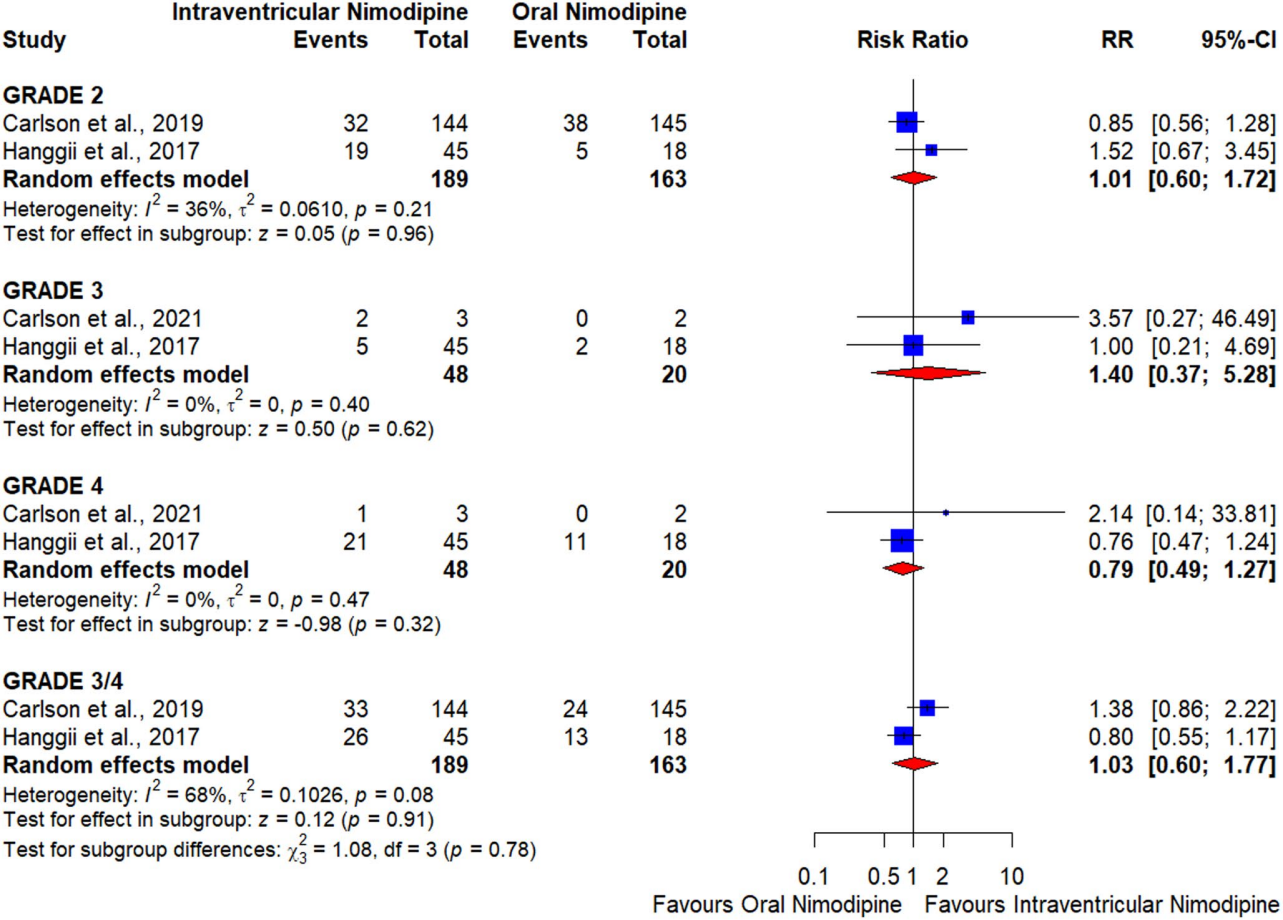
Forest plot shows the favorable functional outcome at day 90 as measured by the extended Glasgow coma scale (eGCS ≥ 6) among people of different World Federation of Neurological Surgeons (WFNS) grades

The pooled effect estimate showed no significant difference between oral and intraventricular nimodipine (RR = 1.01, CI [0.6–1.72], *p* = 0.96) (Fig. 3). The pooled results were homogenous (*p* = 0.21, I^2^ = 36%).

#### WFNS grade 3

The pooled effect estimate showed no significant difference between oral and intraventricular nimodipine (RR = 1.4, CI [0.37–5.28], *p* = 0.62) (Fig. 3). Pooled results were homogenous (*p* = 0.4, I^2^ = 0%).

#### WFNS grade 4

The pooled effect estimate showed no significant difference between oral and intraventricular nimodipine (RR = 0.79, CI [0.49–1.27], *p* = 0.32) (Fig. 3). The pooled results were homogenous (*p* = 0.47, I^2^ = 0%).

#### WFNS grade 3 and 4

The pooled effect estimate showed no significant difference between oral and intraventricular nimodipine (RR = 1.03, CI [0.6–1.77], *p* = 0.91) (Fig. 3). Pooled results were heterogeneous (*p* = 0.08, I^2^ = 68%).

### Angiographic vasospasm

**Fig. 4:**
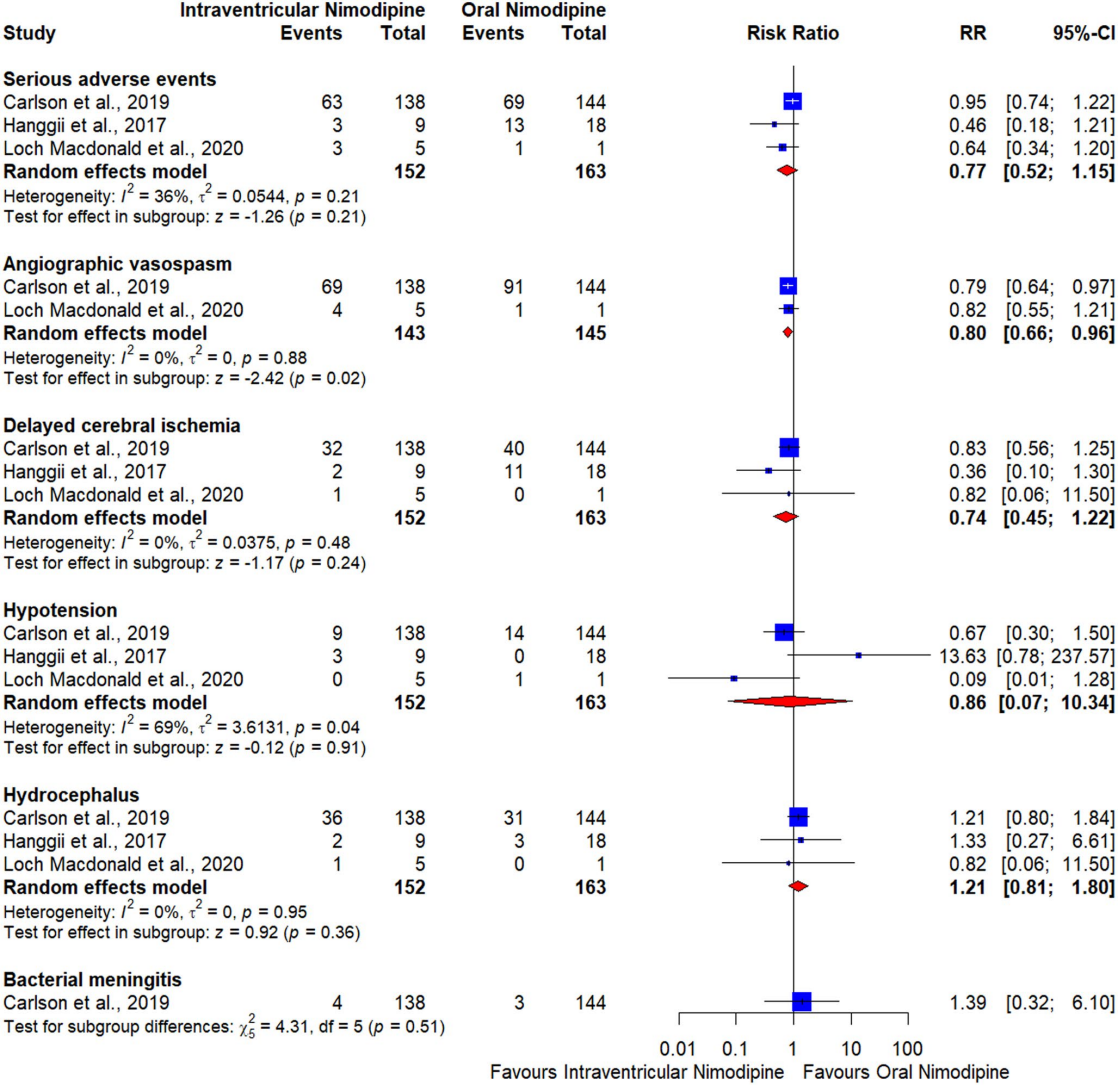
Forest plot shows the secondary outcomes

The pooled effect estimate showed a marginally lower risk of angiographic vasospasm in the intraventricular nimodipine cohort when compared to the oral nimodipine cohort (RR = 0.8, CI [0.66–0.96], *p* = 0.02) (Fig. 4). Pooled results were homogeneous (*p* = 0.88, I^2^ = 0%).

### Delayed cerebral ischemia

The pooled effect estimate showed no significant difference between oral and intraventricular nimodipine (RR = 0.74, CI [0.45–1.22], *p* = 0.24); (Fig. 4). The pooled results were homogeneous (*p* = 0.48, I^2^ = 0%).

### Hypotension

The pooled effect estimate showed no significant difference between oral and intraventricular nimodipine (RR = 0.86, CI [0.07–10.34], *p* = 0.91) (Fig. 4). The pooled results were heterogeneous (*p* = 0.04, I^2^ = 69%).

### Hydrocephalus

The pooled effect estimate showed no significant difference between oral and intraventricular nimodipine (RR = 1.21, CI [0.81–1.80], *p* = 0.36); (Fig. 4). The pooled results were homogeneous (*p* = 0.95, I^2^ = 0%).

### Bacterial meningitis

The pooled effect estimate showed no significant difference between oral and intraventricular nimodipine (RR = 1.39, CI [0.32–6.10], *p* = 0.66) (Fig. 4). Heterogeneity was not applicable.

### Serious adverse events, including death

The pooled effect estimate showed no significant difference between oral and intraventricular nimodipine (RR = 0.77, CI [0.52–1.15], *p* = 0.21) (Fig. 4). The pooled results were homogeneous (*p* = 0.21, I^2^ = 36%).

## Discussion

The findings of our meta-analysis highlight important insights into the comparative efficacy and safety of intraventricular versus oral nimodipine in patients with aSAH. Our results suggest that while intraventricular nimodipine shows a significantly lower risk of angiographic vasospasm compared to oral nimodipine, it did not reach the statistical significance regarding eGCS scores or other adverse events, including delayed cerebral ischemia, hypotension, hydrocephalus, bacterial meningitis, and serious adverse events such as death.

A variety of interventions have been explored for the management of cerebral vasospasm and delayed cerebral ischemia (DCI), including intra-arterial administration of vasodilators such as milrinone, verapamil, and nicardipine. While these agents have shown some promise, robust evidence from phase 3 clinical trials is still lacking [33]. To date, nimodipine remains the only pharmacologic therapy with proven efficacy in improving outcomes following aneurysmal subarachnoid hemorrhage (aSAH) by mitigating the risk of vasospasm and DCI. Other therapeutic approaches include induced hypertension and endovascular balloon angioplasty. However, prophylactic angioplasty has not demonstrated a clear benefit in improving clinical outcomes and carries potential risks, such as arterial rupture, limiting its recommendation in routine preventive use [33].

The meta-analysis found no significant differences in the eGCS by WFNS across all grades between intraventricular and oral nimodipine groups. Specifically, the pooled effect estimates for WFNS grades 2, 3, 4, and combined grades 3 and 4 were not statistically significant. This suggests that, despite a significant reduction in angiographic vasospasm, intraventricular nimodipine does not translate into improved functional outcomes. However, several factors were found to affect eGCS [34–36], and these factors may affect the functional outcomes of the included patients, especially with the long duration of follow-up. The included studies did not take into consideration these factors.

The pooled data indicated a significant reduction in the risk of angiographic vasospasm with intraventricular nimodipine compared to oral administration. This finding aligns with the hypothesized mechanism that direct delivery of nimodipine to the subarachnoid space can more effectively mitigate vasospasm by maintaining higher local drug concentrations near the cerebral arteries, consistent with the pharmacological action of nimodipine in relaxing vascular smooth muscle through L-type calcium channel blockade [29]. Carlson et al. (2021) [32] demonstrated that locally delivered nimodipine microparticles reduced spreading depolarization (SDs), which are closely associated with delayed cerebral ischemia (DCI). This finding suggests that nimodipine may have beneficial effects on delayed cerebral ischemia through two different mechanisms: its impact on SDs and the reduction in large artery vasospasm [37]. The meta-analysis revealed that although intraventricular nimodipine showed less risk of delayed cerebral ischemia, it did not reach statistical significance. This can be explained by the small sample size and the fact that the meta-analysis was based on the NEWTON trials. The Newton-2 trial was a phase 3 trial with the largest sample size. The primary outcome was determined to be the eGCS at 90 days after randomization. However, the trial was early terminated due to a low likelihood of reaching the primary endpoint after an interim analysis of only 210 patients out of 374 patients that were required to meet the power of the study to detect a significant difference between the two groups in terms of eGCS at 90 days. Therefore, the study was not powered to evaluate other critical outcomes that directly represent the beneficial effect of intraventricular nimodipine over oral nimodipine, such as vasospasm, delayed cerebral ischemia, and hypotension. Hänggi et al. (2017) [27] reported no EG-1962-related systemic hypotension cases compared to 17% in the oral nimodipine group.

Therefore, with intraventricular nimodipine having a significantly lower risk of vasospasm and a trend toward a lower incidence of DCI and systemic hypotension than oral nimodipine, there is still a need for future studies with a large enough sample size to confirm these findings, especially when intraventricular nimodipine is given as one single dose compared to oral nimodipine doses that are required to be given every four hours for three weeks. Moreover, it is not uncommon when oral nimodipine-induced systemic hypotension adversely affects cerebral perfusion pressure in patients with cerebral vasospasm, and this prompts neuro-intensivists to reduce oral nimodipine doses to half and sometimes to even a quarter of the regular dose to be given every two hours and one hour, respectively, to prevent the critical drops in cerebral perfusion pressure. These critical situations are extremely challenging and often difficult to manage; therefore, a single dose of intraventricular nimodipine will be a preferable choice over oral nimodipine. Clinicians may consider intraventricular nimodipine for patients who are unable to tolerate oral nimodipine or when the oral regimen would be difficult to implement, given the similar functional outcomes and safety of the two routes. Future studies are still needed before clinical decision-makers recommend intraventricular nimodipine over the oral route.

### Limitations

Our meta-analysis is limited by the small number of included trials, their small population samples, and the potential biases identified in their methodologies. Notably, concerns regarding missing data and potential assessor blinding issues, may overestimate or underestimate the beneficial effects of interventions, highlighting the need for a cautious interpretation of our findings. Publication bias couldn’t be assessed due to small number of the included studies. While RRs are intuitive and commonly used in meta-analyses, they can sometimes mask the absolute effect size, particularly when baseline event rates vary between studies. We also note that although odds ratios (ORs) and risk differences (RDs) offer alternative perspectives, we opted for RRs due to their interpretability and consistency with previous literature in this field.

Future studies should aim to address these methodological limitations and explore the underlying mechanisms contributing to the lack of functional improvement despite the significant reduction in vasospasm, such as the presence of cofounders. Additionally, future research should focus on identifying other therapeutic targets and adjunctive vasospasm therapy, such as intra-arterial administration of vasodilators such as milrinone, verapamil, and nicardipine, that may improve functional outcomes and reduce the overall morbidity and mortality associated with aSAH.

## Conclusion

Intraventricular nimodipine had a lower risk of angiographic vasospasm than oral nimodipine, as well as a trend toward a reduced incidence of DCI and systemic hypotension, which should be confirmed in future studies. However, there is no substantial improvement in functional outcomes with intraventricular nimodipine. More rigorous research is needed to investigate the underlying mechanisms and determine whether other factors influence this outcome, including the possibility of higher doses or more than one administration of intraventricular nimodipine.

## Electronic supplementary material

Below is the link to the electronic supplementary material.

Supplementary Material 1

## Data Availability

No datasets were generated or analysed during the current study.
